# Nanoparticle Mediated P-Glycoprotein Silencing for Improved Drug Delivery across the Blood-Brain Barrier: A siRNA-Chitosan Approach

**DOI:** 10.1371/journal.pone.0054182

**Published:** 2013-01-23

**Authors:** Jostein Malmo, Axel Sandvig, Kjell M. Vårum, Sabina P. Strand

**Affiliations:** 1 Norwegian Biopolymer Laboratory (NOBIPOL), Department of Biotechnology, Norwegian University of Science and Technology (NTNU), Trondheim, Norway; 2 Department of Laboratory Medicine, Children's and Women's Health, Norwegian University of Science and Technology (NTNU), Trondheim, Norway; 3 MI lab and Department of Circulation and Medical Imaging, Norwegian University of Science and Technology (NTNU), Trondheim, Norway; 4 Department of Neurosurgery, Umeå University Hospital, Umeå, Sweden; Arizona State University, United States of America

## Abstract

The blood-brain barrier (BBB), composed of tightly organized endothelial cells, limits the availability of drugs to therapeutic targets in the central nervous system. The barrier is maintained by membrane bound efflux pumps efficiently transporting specific xenobiotics back into the blood. The efflux pump P-glycoprotein (P-gp), expressed at high levels in brain endothelial cells, has several drug substrates. Consequently, siRNA mediated silencing of the P-gp gene is one possible strategy how to improve the delivery of drugs to the brain. Herein, we investigated the potential of siRNA-chitosan nanoparticles in silencing P-gp in a BBB model. We show that the transfection of rat brain endothelial cells mediated effective knockdown of P-gp with subsequent decrease in P-gp substrate efflux. This resulted in increased cellular delivery and efficacy of the model drug doxorubicin.

## Introduction

Gene silencing by RNA-interference (RNAi) is a relatively new technology with potential to revolutionize medicine by offering specific deactivation of genes in mammalian cells [1]. RNAi can be mediated by intracellular delivery of siRNA (short interfering RNA) duplexes that binds specifically to complementary mRNA sequences, leading to degradation of the target mRNA and inhibition of protein synthesis. siRNA is a polyanionic molecule of approximately 13 kDa which is rapidly degraded by RNases. These intrinsic properties of siRNA make the delivery into mammalian cells a challenge, currently limiting the implementation of this technology into the clinic [2]. To improve the cellular delivery of siRNA, several delivery vehicles based on lipids [1], [3] and cationic polymers [4]–[7] have been developed. Upon mixing with siRNA, the cationic polymers form spontaneously nanoparticles with siRNA. Among the polymer derived nanoparticles, most research has been done on delivery vehicles based on polyethyleneimine [5], [6] and chitosan [4], [7].

Chitosan is a cationic biopolymer derived from chitin, which is one of the most abundant biopolymers on Earth [8]. In contrast to most polycations, chitosan has an excellent biocompatibility, low toxicity (reviewed in [9]) in addition to being biodegradable [10], [11]. Chitosan is chemically composed of β-(1,4) linked monomers of *N*-acetylated D-glucosamine and positively charged D-glucosamine units, and can be prepared with widely varying fraction of *N*-acetylated units (F_A_) and chain lengths (DP_n_). Chitosan can be considered as a family of polysaccharides with very different functional properties [8]. Therefore, the properties and efficiency of chitosan-based delivery systems for nucleic acids are strongly dependent on the structure of chitosan. For instance, it has been shown that the optimal chitosan for DNA delivery are different from those required for siRNA delivery [4], [12]–[14]. The molecular properties of chitosan essential for efficient delivery of siRNA into mammalian cells have recently been characterized, showing that more high molecular weight chitosans are required for efficient for delivery of siRNA as compared to DNA [4], [12]–[14]. We have shown that nanoparticles based on fully de-*N*-acetylated chitosans of DP_n_>50 mediated approximately 90% gene silencing of the target gene even at low siRNA concentrations and without toxic effects [4].

The blood-brain barrier (BBB) constitutes an efficient organization of tight junctions between endothelial cells in the brain tissues (reviewed in [15]). This barrier prevents paracellular entry of harmful substances into the brain interstitium and protects cells in the central nervous system (CNS). In addition, the cellular efflux pumps form another layer of defence and maintains the BBB by efficient excretion of specific xenobiotics diffused into or taken up by the endothelial cells [15]. Consequently, drugs are transported across the BBB at very low efficiency, and this currently limits the treatment of e.g. schizophrenia [16], depression [17], brain tumors [18], HIV [19]and epilepsy [20]. The best characterized drug efflux pump is P-glycoprotein (P-gp), involved in several anatomical and physiological barriers [21]–[25] and also in cancer cell drug resistance [26]–[29]. Previous work on strategies to avoid P-gp mediated drug efflux at the BBB includes the use of specific inhibitors [30], altering the gene regulation [31] and lipid mediated drug transport to increase the cellular uptake [32]. In addition, repeated injections of naked siRNA in mice *in vivo* has recently been shown to significantly reduce the expression of P-gp in brain endothelial cells [33].

In this work, we have investigated whether siRNA-mediated silencing of P-gp lead to improved drug delivery in an *in vitro* BBB model. First, we evaluated the siRNA-chitosan nanoparticle uptake and transfection efficiency in RBE4 cells; a cell line of endothelial origin derived from rat brain tissue and commonly used as a BBB model [34], [35]. Following the knockdown of P-gp in the RBE4 cells we studied whether the silencing lead to reduced efflux and increased intracellular accumulation of the P-gp substrates rhodamine 123 (R123) and doxorubicin, used herein as model drugs. We show that P-gp silencing using chitosan-siRNA nanoparticles resulted in improved delivery and efficacy of doxorubicin, indicating that this strategy can be suitable to improve the drug delivery into the CNS.

## Materials and Methods

A more detailed description of the materials and methods can be found in Supporting Information S1.

### siRNA

The following siRNA sequences used in this study were predesigned and supplied by Ambion: anti-P-gp (Silencer Select, sense 5′-GCUGGUAUUUGGGCAAAGAtt-3′, antisense 5′-UCUUUGCCCAAAUACCAGCtg-3′), anti-GAPDH (Silencer Select) in addition to a non-targeting (NT) siRNA sequence (Silencer Select, Negative Control #1). For flow cytometry and confocal microcscopy (CLSM), a NT Alexa-647 conjugated siRNA duplex (AllStars Negative Control, Qiagen) was used.

### Chitosan

The fully de-*N*-acetylated chitosan (F_A_<0.002) used in this study was prepared in our laboratory from a commercial chitosan with F_A_ 0.01 (Pronova Biopolymers) by heterogenous de-*N*-acetylation, as previously described [36]. The chitosan characteristics are listed in Table 1.

**Table 1:** Molecular characterization of the chitosan used in the study.

DP_n_	M_n_	M_w_	PDI	F_A_
	kDa	kDa		
375	75.1	203	2.7	<0.002

The weight and number average of the molecular weight (M_w_, M_n_) and the polydispersity index (PDI) were analyzed by SEC-MALLS. The fraction of *N*-acetylated units (F_A_) was determined by ^1^H NMR.

### Preparation of siRNA-chitosan nanoparticles

Formulations with different amino/phosphate (N/P) ratios were prepared by a self-assembly method. A solution of siRNA was diluted with sterile nuclease free water (5 Prime). Subsequently, chitosan was added from a sterile solution during vortex mixing. The assembled nanoparticles were incubated for 30 min at room temperature before transfection.

### Nanoparticle tracking analysis

The nanoparticle concentrations were determined using nanoparticle tracking analysis (NTA) on a NanoSight LM10 (NanoSight) at a siRNA concentration of 500 nM. Measurements were performed in MQ water at room temperature using the viscosity of water in the calculations. The CCD camera was operated and video was captured with the software NTA 2.0.

### Cell culture

The immortalized rat endothelial cell line RBE4 [35] was kindly provided by Prof. Tore Syversen (Dept. of Neuroscience, NTNU). The cells were grown in alpha MEM (aMEM, Gibco, Invitrogen) supplemented with 10% FBS, 300 µg/mL G418 selection antibiotic (Sigma) and 1 ng/mL basic fibroblast growth factor (Invitrogen). When seeding cells for experiments, growth media supplemented with 100 U/mL of penicillin and streptomycin (PEST, Sigma) was used. The cells were cultured on surfaces coated with rat tail type I collagen (BD Biosciences) at 37°C in a humidified atmosphere with 5% CO_2_.

### Transfection

Cells were seeded in tissue culture wells (Corning) 24 h prior to experiments in densities with approximately 50–75% confluency on the day of transfection. The nanoparticles assembled in water were diluted with an equal volume of Opti-MEM (Gibco, Invitrogen), supplemented with 270 mM mannitol (Sigma) and 20 mM HEPES (Sigma) for adjustment of the osmolarity to 300 mOsm/kg and the pH to 7.2. Prior to adding the nanoparticles, the cells were washed and briefly incubated with Hank's balanced salt solution (HBSS, Gibco, Invitrogen) at 37°C and 5% CO_2_. Next, the HBSS solution was removed and nanoparticle formulations were added to each well in 96-well plates. The formulations were removed after 5 h of incubation and replaced by growth media supplemented with PEST.

### Rhodamine 123 efflux assay

Two days after transfection with anti-P-gp siRNA, cells were incubated with the P-gp substrate rhodamine 123 (R123, Sigma). A 10 µM R123 solution diluted in Opti-MEM was added to the cells. After 45 min of incubation, R123 was removed and replaced with growth medium. Two hours after removing the R123, cells were prepared for analysis by flow cytometry or confocal laser scanning microscopy (CLSM).

### Doxorubicin delivery and metabolic activity assay

One day after transfection with anti-P-gp siRNA, the RBE4 cells were added growth medium with concentrations of the P-gp substrate, and DNA intercalating agent, doxorubicin (Pharmacia) ranging from 0 to 5 µM. The cells were incubated with doxorubicin for two days before the effect on metabolic activity was measured using an Alamar Blue assay (Invitrogen). The Alamar Blue assay reagent diluted in growth medium without phenol red was added to the cells and the sample absorbances were measured 4 h after adding the assay reagent using a spectrophotometer (Molecular Devices) at 570- and 600-nm. The metabolic activities of the cells were determined from the fraction of Alamar Blue reagent that was turned over during a 4 h incubation period.

The evaluation of intracellular doxorubicin delivery by flow cytometry and CLSM was performed by incubating the cells in growth medium with 50 µM doxorubicin for 3 h before analysis.

### Flow cytometry

Cellular uptake of siRNA, R123 efflux and doxorubicin delivery were evaluated using a Gallios flow cytometer (Beckman Coulter). The obtained data were analyzed and visualized using the Kaluza software package (Kaluza Flow Cytometry Analysis v1.1, Beckman Coulter).

The cellular uptake of siRNA was determined by transfection with Alexa-647 conjugated siRNA. After incubating with nanoparticles for 4 h, the cells were washed with PBS and further incubated with aMEM for 30 min and heparin supplemented aMEM (1 mg/mL, Sigma) for another 30 min. The cells were then washed in PBS (Gibco, Invitrogen), trypsinized, resuspended in ice-cold PBS supplemented with 5% FBS and kept on ice until the time of analysis.

Intracellular R123 and delivery of doxorubicin was measured 48 h after transfection with anti-P-gp siRNA as previously described.

The R123 and doxorubicin or Alexa-647 treated cells were excited using a 488 nm or 633 nm laser line, respectively. Emitted light was collected at FL1 (R123), FL2 (doxorubicin) or FL6 (Alexa-647) using 525/40 nm, 575/40 nm or 660/20 nm band pass filter, respectively. The relative amounts of intracellular Alexa-647, R123 or doxorubicin were estimated from the median FI of the analyzed cells.

### Real-time quantitative reverse transcriptase PCR

Knockdown of the ubiquitously expressed endogenous gene GAPDH (Glyceraldehyde-3-phospate dehydrogenase) and P-gp was measured at mRNA level using the ABI 7500 real-time PCR system (Applied Biosystems). The mRNA was harvested, and cDNA was synthesized and amplified using the Cells-to-C_T_ kit (Applied Biosystems) as described in the manufacturer's protocol. Reverse transcription was performed at 37°C for 60 min. Real-time quantitative reverse transcriptase PCR (qRT-PCR) was performed using the following cycle conditions: 95°C for 10 min, 40 cycles at 95°C for 15 s and 60°C for 1 min. The primers that were used are described in Table 2.

**Table 2:** The qRT-PCR primers used in the study.

Primer	Direction	Sequence	Supplier
target		(5′-3′)	
GAPDH	Forward	TCGGTGTGAACGGATTTG	MWG Operon
GAPDH	Reverse	CCGTGGGTAGAGTCATACTGG	MWG Operon
P-gp	Forward	AGCCCTGTTCTTGGACTG	Sigma
P-gp	Reverse	AGTTCTGATGGCTGCTAAGAC	Sigma
β-actin	Forward	TCCACCTTCCAGCAGATGTG	MWG Operon
β-actin	Reverse	GCATTTGCGGTGCACGAT	MWG Operon

The primer efficiencies were determined using standard curves. The percentage of mRNA expression relative to untreated cells was calculated using the comparative C_t_ method, where the target sample was normalized to endogenous β-actin.

### GAPDH protein activity assay

The effect of transfection with anti-GAPDH siRNA on the GAPDH protein activity was measured using the commercial available KDalert GAPDH assay kit (Ambion) according to the manufacturer's protocol. The amounts of lysate and assay reagents were halved, and measurements were performed in half-area 96-well plates (Corning) at 615 nm using a spectrophotometer.

### CLSM

RBE4 cells were seeded onto type I collagen coated 8-chamber microscopic slides (Ibidi) and transfected with Alexa-647 labeled siRNA or anti-P-gp siRNA with subsequent addition of R123 or doxorubicin as described previously. At the time of analysis, the cells were added 5 µg/mL of CellMask plasma membrane stain (Invitrogen) diluted in aMEM, as described in the manufacturer's protocol. Live cells were examined using a LSM 510 (Carl Zeiss) confocal laser scanning microscope equipped with a c-Apochromat 40×/1.2 NA W corr objective. R123 and doxorubicin were excited using 488 nm argon, CellMask Orange was excited using 543 nm HeNe and Alexa-647 and CellMask Deep Red were excited using a 633 nm HeNe laser line. The emitted light was collected using 525/25 nm band pass (R123), 590/25 band pass (CellMask Orange and doxorubicin) or 650 nm long pass (CellMask Deep Red) filters. The acquired images had resolutions of 512×512 pixels.

### Statistical analysis

The measurements were collected and expressed as mean values ± standard deviation (s.d.). Statistical differences between raw data were investigated using the SigmaPlot 11.0 software package with one-way ANOVA in conjunction with a multiple comparison test (Holm-Sidak).

## Results

### Transfection of the RBE4 cell line

The aim of this study was to determine whether increased drug delivery to brain endothelial cells could be obtained by silencing P-gp expression using siRNA-chitosan nanoparticles. The nanoparticles were assembled from a fully de-*N*-acetylated chitosan of intermediate chain length (Table 1) that we have previously found to be optimal for siRNA delivery and transfection in mammalian cells [4]. The nanoparticles formed using this chitosan were evaluated for their ability to transfect the RBE4 cell line, commonly used as a BBB model.

**Figure 1:**
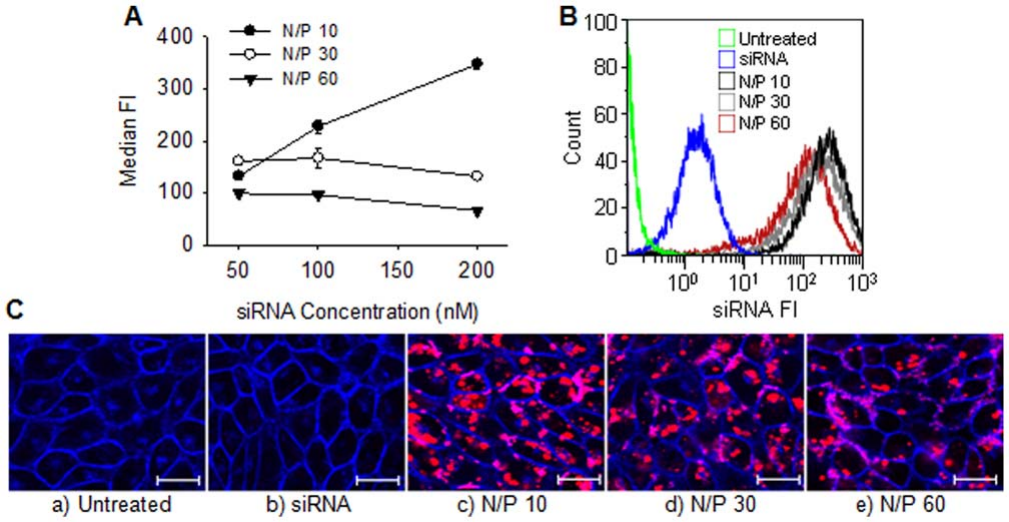
Chitosan-mediated siRNA uptake in RBE4 cells. A) Levels of internalized Alexa-647 conjugated siRNA at different nanoparticle N/P ratios and siRNA concentrations expressed as the median fluorescence intensities (FI) of the analyzed cells. Data represents mean values ± s.d., n = 3. B) Representative histograms of siRNA fluorescence from flow cytometry analysis of untreated cells, cells with added naked siRNA or transfected with nanoparticles having N/P 10, 30 or 60 and a siRNA concentration of 100 nM. C) Representative CLSM images of a) untreated cells, b) cells with added naked siRNA or nanoparticles having N/P c) 10, d) 30 or e) 60 and a siRNA concentration of 100 nM. The cellular plasma membrane was stained with CellMask Orange (blue) and the fluorescent siRNA is indicated with the red color. The bar size is 20 µm.

Figure 1A-C shows the internalization of Alexa-647 conjugated siRNA nanoparticles with varying amino/phosphate (N/P) ratio. The amount of internalized siRNA depended both on the N/P ratio of the formulation and the concentration of siRNA. As shown in Figure 1A, formulations with a N/P ratio of 10 showed increased uptake with increasing siRNA concentration whereas the formulations with N/P ratios of 30 and 60 showed relatively stable uptake, independent on the siRNA concentrations. Surprisingly, at a constant siRNA concentration of 100 nM, the amount of internalized siRNA decreased with increasing N/P (Figure 1A and B) as also confirmed from the CLSM images shown in Figure 1C.

**Figure 2:**
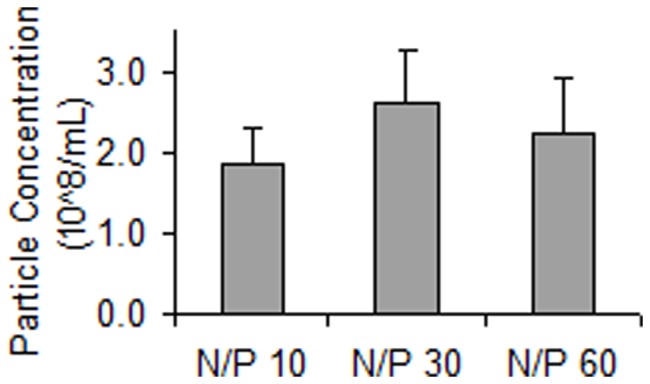
Particle concentrations measured by nanoparticle tracking analysis. The samples consisted of complexes in formulations having N/P 10, 30 or 60 and a siRNA concentration of 500 nM. Data represents mean values ± s.d., n = 3.

Since the uptake of siRNA nanoparticles depended on N/P ratio of the formulation, we measured the nanoparticle concentrations as a function of NP ratio by nanoparticle tracking analysis to determine the amount of particles per volume of the formulations. However, as shown in Figure 2, the particle concentrations were not significantly different at the varying N/P ratios with between 2 and 2.5•10^8^ particles/mL in the three formulations.

**Figure 3:**
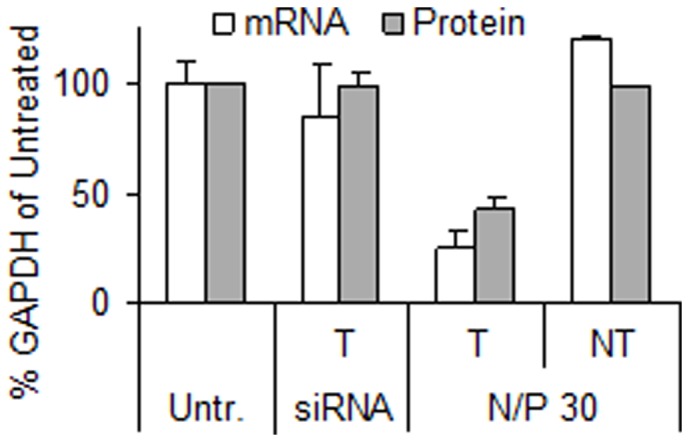
Knockdown of GAPDH measured by levels of mRNA and protein activity. The nanoparticles had a N/P ratio of 30 and a concentration of 50 nM GAPDH targeting (T) or non-targeting (NT) siRNA. Cells were also treated with naked siRNA (siRNA). Data represents mean values ± s.d., n = 3.

The knockdown efficiency of the nanoparticles was determined using anti-GAPDH siRNA targeting the ubiquitously expressed endogenous gene GAPDH in the RBE4 cells. Preliminary experiments revealed that the highest knockdown efficiency was obtained with nanoparticle formulations with a N/P of 30 or higher (data not shown), despite lower uptake compared to formulations with a N/P of 10. Therefore, further experiments were performed with nanoparticles with N/P of 30. The results in Figure 3 show that levels of both GAPDH mRNA and protein activity are reduced two days post-transfection to approximately 25 and 40% relative to the untreated cells, respectively. The transfection with naked siRNA or chitosan-formulated NT siRNA resulted in levels of GAPDH comparable to the untreated cells, indicating that the nanoparticles were non-toxic.

### P-gp silencing efficiency and the effect on substrate efflux

**Figure 4:**
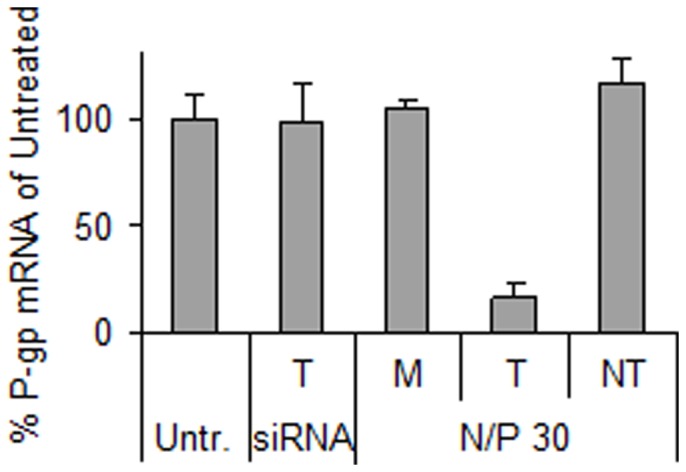
Knockdown of P-gp measured at mRNA level by qRT-PCR. The cells were transfected with only chitosan (mock, M) or nanoparticles having N/P 30 and P-gp targeting (T) or non-targeting (NT) siRNA concentrations of 100 nM. Cells were also treated with naked siRNA (siRNA). Data represents mean values ± s.d., n = 3.

Following confirmation that the siRNA-chitosan nanoparticles were able to efficiently transfect the RBE4 cells, the particles were assembled with anti-P-gp siRNA to silence the P-gp drug efflux pump. As shown in Figure 4, the transfection resulted in reduced P-gp mRNA levels to approximately 20% compared to the untreated cells. In addition, no significant change in mRNA expression was observed from the mock transfection with chitosan (M) or NT siRNA delivery, indicating absence of non-specific effects from the chitosan or nanoparticles, respectively.

**Figure 5:**
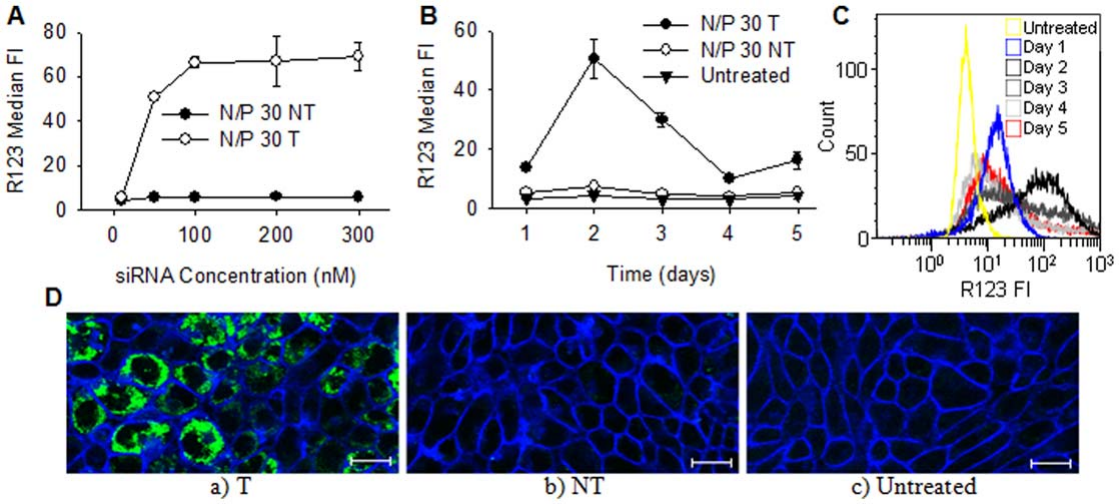
The effect of P-gp knockdown on R123 efflux. Intracellular levels of R123 as a function of A) siRNA concentration and B) days post-transfection. The relative levels of R123 are expressed as the median FI of the cells. The cells were transfected with nanoparticles having N/P 30 and P-gp targeting (T) or non-targeting (NT) siRNA concentrations of 100 nM. Data represents mean values ± s.d., n = 3. C) Representative histograms of R123 fluorescence from flow cytometry analysis of untreated cells or cells transfected with nanoparticles having N/P 30 and a siRNA concentration of 100 nM at one to five days post-transfection. D) Representative CLSM images after incubation with R123 post-transfection with a) T or b) NT siRNA or c) untreated cells. The cells were transfected with nanoparticles having N/P ratios of 30 and a siRNA concentration of 100 nM. The cellular plasma membranes were stained with CellMask Deep Red (blue) and R123 fluorescence is indicated with the green color. The bar size is 20 µm.

Next, the fluorescent P-gp substrate R123 was used as a molecular marker to investigate the reduction in cellular efflux in the transfected cells. Cells were transfected using formulations of different siRNA concentrations in order to investigate the effect of the siRNA concentration on the degree of P-gp mediated efflux. The results are given in Figure 5A, showing an extensive increase in intracellular R123 when the siRNA concentration was increased to and above 50 nM. The amount of accumulated R123 peaked when the cells were transfected with 100 nM siRNA, and there was no additional effect when further increasing the concentration. The transfection with NT siRNA resulted in low levels of intracellular R123, similar as for the untreated cells (data not shown). Based on these results, a concentration of 100 nM siRNA was used to minimize P-gp mediated substrate efflux by gene silencing.

To characterize the P-gp knockdown kinetics in the RBE4 cells, R123 efflux in transfected cells were monitored for up to five days. The results presented in Figure 5B and C show that after an initial weak effect one day post-transfection, the maximum accumulation of R123 was recorded after two days. The efflux then increased, but the level of intracellular R123 remained high until day four when it returned to similar level as one day post-transfection. If longer silencing is required, it is possible to further reduce the substrate efflux and increase the duration of knockdown by repeated transfections (data not shown). The intracellular distribution of R123 is visualized by CLSM in Figure 5D, showing a clear accumulation of R123 in the majority of the transfected cells. In contrast, the untreated cells and cells transfected with NT siRNA were R123 negative, confirming the functional efflux mediated by P-gp.

### The effect of P-gp silencing on drug efficacy and delivery

**Figure 6:**
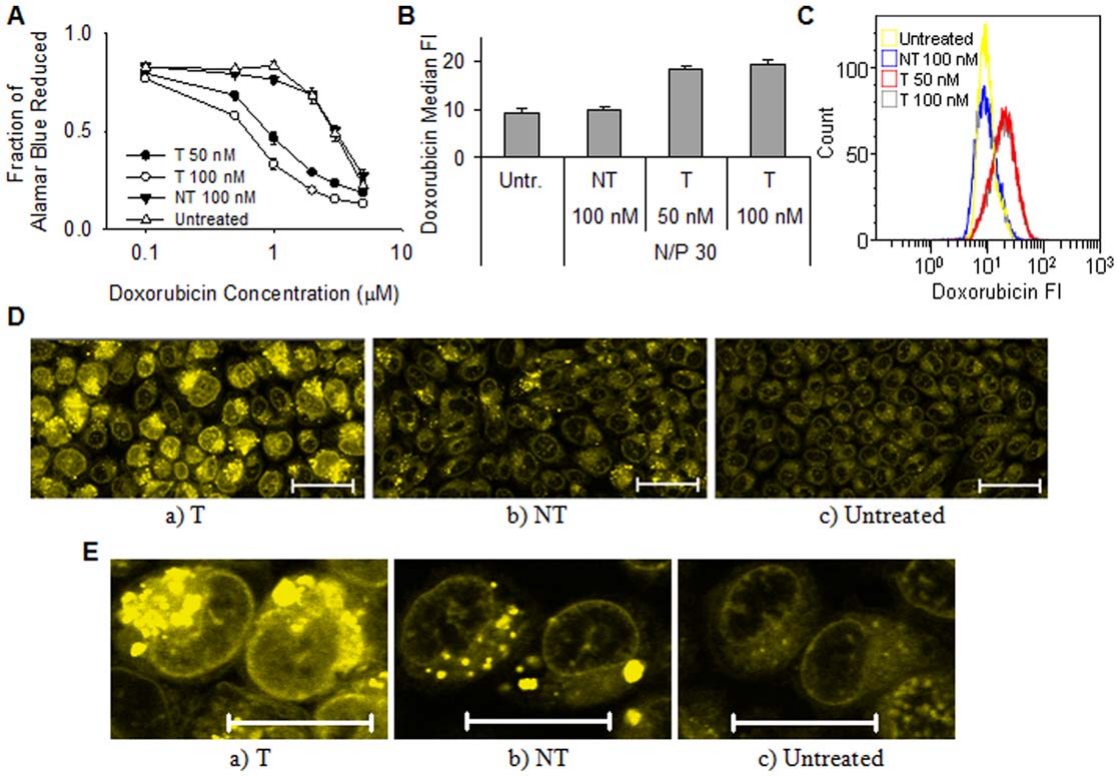
The effect of P-gp knockdown on doxorubicin efficacy and delivery. A) Metabolic activity of cells after two days of incubation with doxorubicin. The cells were transfected with nanoparticles having N/P 30 and P-gp targeting (T) or non-targeting (NT) siRNA concentrations of 50 or 100 nM. Data represents mean values ± s.d., n = 4. B) Intracellular uptake and accumulation of doxorubicin expressed as the median FI of the cells. The cells were transfected with nanoparticles having N/P 30 and T or NT siRNA concentrations of 100 nM. Data represents mean values ± s.d., n = 3. C) Representative histograms of doxorubicin fluorescence from flow cytometry analysis of untreated or transfected cells. D) Representative CLSM images after transfection with a) T or b) NT siRNA or c) untreated cells. E) Enlarged images of RBE4 nuclei. The cells were transfected with nanoparticles having N/P 30 and a siRNA concentration of 100 nM. Doxorubicin fluorescence is indicated with the green color. The bar size is 20 µm.

To determine the effect of increased intracellular delivery of P-gp substrate drugs on the cellular physiology, differences in metabolic activities after treatment with the DNA intercalating agent doxorubicin was evaluated. As shown in Figure 6A, cells with P-gp knocked down showed considerably higher sensitivity to doxorubicin, even at doxorubicin concentrations as low as 0.5 µM. The efficacy of doxorubicin treatment was even higher at 1 µM, where the cells transfected with anti-P-gp siRNA at 100 nM showed a 60% reduction in metabolic activity compared to the cells with normal P-gp expression. A further increase in the concentration of doxorubicin also resulted in a reduction of the metabolic activity in the untreated cells. The cells showed higher sensitivity to doxorubicin when they were transfected with siRNA concentrations of 100 nM as compared to 50 nM (Figure 6A). Furthermore, the effect of doxorubicin on the metabolic activity after transfecting with NT siRNA at 100 nM was similar as for the untreated cells. When no doxorubicin was added to the growth medium, the transfected cells were as metabolically active as the untreated cells, indicating that the nanoparticles were non-toxic (data not shown).

The intracellular delivery of doxorubicin was further evaluated by flow cytometry as shown in Figure 6B and C. The measured median FI values were doubled in cells transfected with anti-P-gp siRNA as compared to the untreated or NT transfected cells. The delivery of doxorubicin was also visualized by CLSM (Figure 6D and E). The CLSM images show an apparently homogenous cytoplasmic distribution of doxorubicin both in the untreated and NT transfected cells. However, the images also show that doxorubicin was only able to accumulate and to intercalate with DNA in the nucleus of cells where P-gp was silenced.

## Discussion

The xenobiotic efflux pump P-gp is expressed in cells at the anatomical and physiological barriers in mammalian tissues and also in malignant cells [21]–[26]. Several of the identified P-gp substrates are drugs [37] and consequently a considerable research effort has focused on finding ways to overcome drug efflux from P-gp expressing cells. Temporary silencing of the P-gp gene by RNAi is a possible way to inhibit the efflux, and this approach has been applied to overcome drug resistance in cancer cells by improving the delivery of chemotherapeutic agents [27]–[29], [38]. Recently, a preliminary study showed a reduction in P-gp expression in brain endothelial cells *in vivo* by repeated hydrodynamic injections of naked siRNA intravenously in mice [33], but extremely high doses of siRNA were used in this study. We hypothesized that the delivery of siRNA formulated in nanoparticles may represent a more rational approach as the nanoparticles will protect siRNA from degradation and facilitate the uptake, thereby allowing the use of lower doses. The naturally derived biopolymer chitosan has been chosen as a delivery vehicle for anti-P-gp siRNA primarily due to its favourable safety profile which is an essential prerequisite for drug delivery into the CNS. We demonstrate herein that siRNA-chitosan nanoparticles can efficiently silence the P-gp gene expression in rat brain endothelial cells which leads to reduced substrate efflux and improved drug delivery.

The chitosan mediated efficient uptake of siRNA by the RBE4 cells (Figure 1A–C). As shown in Figure 1A, the degree of nanoparticle uptake depended on the N/P ratio. Since the formulations prepared at different N/P ratios contained similar number of particles per volume (Figure 2), and were of similar size (data not shown), it can be assumed that the higher the N/P ratio, the higher is the excess of chitosan in the formulation. The excess of free unbound chitosan at higher N/P ratios may inhibit the uptake of siRNA by binding to cellular surfaces and preventing the attachment of siRNA-chitosan nanoparticles. This is consistent with the increased siRNA uptake observed at the lower N/P ratios. However, despite higher uptake, nanoparticles with low N/P ratios showed lower transfection efficacy (data not shown). This may be related to low stability of these nanoparticles and premature intracellular dissociation of siRNA [39]–[41]. Thus, we chose the intermediate N/P ratio of 30 for the assembly of the nanoparticles in this study.

The gene expression analysis of GAPDH and P-gp shown in Figure 3 and 4, respectively, confirmed the ability of the siRNA-chitosan nanoparticles to efficiently silence genes in the RBE4 cell line with a reduction in mRNA levels of approximately 80% compared to the untreated cells. Chitosans have repeatedly and by independent groups been shown to mediate efficient nucleic acid delivery *in vitro*, and is often the gene delivery vehicle of choice [4], [13], [14], [42]. Furthermore, the NT siRNA and mock transfections indicated that there were no side-effects from the transfections affecting the gene expression or the viability of the cells. This is in agreement with several studies illustrating that chitosan is a siRNA delivery agent with low cytotoxicity [4], [39], [41].

The concentration-response curve illustrated in Figure 5A shows a considerably lower R123 efflux as the concentration of siRNA increased from 10 to 100 nM. A concentration of 50 nM has been previously reported sufficient for gene knockdown when using fully de-*N*-acetylated chitosans as delivery vehicles [4]. However, this may depend on the cell line and the expression of the target gene. The P-gp gene is known to be relatively weakly expressed in the RBE4 cell line [43] and this was confirmed by qRT-PCR in our study (data not shown). Silencing a weakly expressed gene could require a higher concentration of siRNA before the effect is observed due to strong regulation and low availability of target mRNA [44], [45]. This was supported by experiments with transfection of C6 cells with even weaker expression of P-gp compared to the RBE4 cells (data not shown). In this case, efficient silencing of P-gp was not achieved despite promising preliminary GAPDH silencing experiments at low siRNA concentrations. Furthermore, the knockdown kinetics presented in Figure 5B and C show that the reduced P-gp mediated efflux lasted only from one to four days post-transfection. Such short duration of P-gp knockdown has also been observed in other studies with different cancer cell lines, where the protein expression was shown to reach its lowest levels one to two days after transfection, and recovered after two to three days [28], [29]. A short duration of P-gp down-regulation is beneficial since it allows rapid re-establishment of the protective function of the BBB after drug therapy. Although the maximum P-gp knockdown was observed two days post-transfection, a maximum reduction in P-gp mRNA was accomplished already one day after transfection. The delay in effect on P-gp efflux is probably caused by the relative long half-life of the P-gp protein, reported to be 14–17 h [46].

The reduction in P-gp mediated efflux following successful siRNA transfection improved the delivery and considerably increased the efficacy of doxorubicin (Figure 6). As shown in Figure 6A the cells were more sensitive to doxorubicin when they were transfected with 100 nM anti-P-gp siRNA than 50 nM. On the other hand, it is apparent from Figure 6B and C that comparable amounts of doxorubicin were internalized at both concentrations of siRNA. Probably, the cells have been saturated with the dose of doxorubicin (50 µM) used in the flow cytometry and CLSM experiment, where a higher dose was used to visualize the intracellular doxorubicin. Consequently, no detectable differences in doxorubicin delivery are observed. In contrast, the relatively low doses of doxorubicin used in the metabolic activity assay are unable to saturate the cells and will be efficiently effluxed or accumulated, depending on the degree of achieved knockdown. As shown in Figure 6D and E, doxorubicin was located intracellularly both in transfected and non-transfected cells. However, the drug was only able to intercalate with DNA in the nucleus after P-gp knockdown. This suggests that P-gp is located both at the cellular membrane, as also indicated from the R123 experiments (Figure 5), and at the nuclear envelope. Indeed, the expression and localization of P-gp in RBE4 cells have previously been confirmed at both sites [47]. Furthermore, doxorubicin has been shown to depend on P-gp silencing for delivery to the nucleus in the multi-drug resistant cell line KB-V1 [27]. This illustrates that an improved delivery of drugs does not necessarily enhance their efficacy, as efflux pumps can still prevent them from reaching their final destination, such as the nucleus in the case of doxorubicin. Similarly, the dose of drugs needed to obtain a therapeutic window could be considerably reduced if a larger fraction reaches its target, which in turn will reduce potential deleterious side effects from the drug.

Any P-gp substrate can also be a substrate for other drug efflux pumps, e.g. R123 has been reported to be transported by Mrp1 [48], which is also expressed in RBE4 cells [43]. Therefore, even though the P-gp is successfully downregulated, efflux by other pumps may still occur. In addition, P-gp is encoded by two different genes in rodents, *mdr1a* and *mdr1b*, with partly overlapping substrate specificity and efflux efficiency [49]. In this study we focused on delivering siRNA targeting *mdr1a*, as we could not measure any effect on R123 efflux when silencing *mdr1b* (data not shown). Despite the possibility of having to deal with several different drug efflux pumps to improve the delivery of a certain drug, this possibility could be solved by assembling nanoparticles with a pooled library of siRNAs targeting several different mRNA sequences.

## Conclusions

We show that siRNA-chitosan nanoparticles are able to efficiently silence the P-gp gene in a BBB model. The knockdown resulted in a considerable reduction in P-gp substrate efflux and improved delivery and efficacy of doxorubicin, which we used as a model drug. Our results suggest that a nanoparticle mediated delivery of anti-P-gp siRNA could be a feasible approach to improve the treatment of various diseases in the CNS where drug delivery is currently limited by the BBB.

## Supporting Information

Supporting Information S1 **Supplementary methods.**
(DOC)
