# Supplementary material for: Nanoparticle Mediated P-Glycoprotein Silencing for Improved Drug Delivery across the Blood-Brain Barrier: A siRNA-Chitosan Approach
Source: PLoS One. 2013 Jan 23;8(1):e54182. doi: 10.1371/journal.pone.0054182 (PMC3553124; doi:10.1371/journal.pone.0054182)
Supplement: Supporting Information S1 — Supplementary methods. (DOC) [file pone.0054182.s001.doc]

**Supplementary methods**

*siRNA*

The following siRNA sequences used in this study were predesigned and supplied by Ambion: anti-P-gp (Silencer Select, sense 5’-GCUGGUAUUUGGGCAAAGAtt-3’, antisense 5’-UCUUUGCCCAAAUACCAGCtg-3’), anti-GAPDH (Silencer Select) in addition to a non-targeting (NT) siRNA sequence (Silencer Select, Negative Control #1). For flow cytometry and confocal microcscopy (CLSM), a NT Alexa-647 conjugated siRNA duplex (AllStars Negative Control, Qiagen) was used.

*Chitosan*

The chitosan oligomer used in this study was prepared in our laboratory from shrimp chitin by nitrous acid depolymerization and NaBH_4_ reduction of a fully de-*N*-acetylated chitosan (F_A_<0.002), as previously described [1]. The chitosan characteristics are described in Table 1 (manuscript).

To determine the F_A_, the chitosan sample was characterized by ^1^H NMR (Avance DPX 400, Bruker). The molecular weight and the polydispersity distribution were determined by size-exclusion chromatography (SEC) with a refractive index detector (RI, Dawn Optilab 903, Wyatt Technology) and a multiangle laser light scattering detector (MALLS, Dawn DSP, Wyatt Technology). Samples were dissolved in Milli-Q (MQ) deionized water (5-7 mg/mL) and filtered through a 0.22 μm syringe filter (Millipore). A TSK 3000 PWXL column (Tosoh Bioscience) was used, and the sample was eluted with 0.2 M ammonium acetate (pH 4.5) at a low flow rate of 0.5 mL/min.

*Preparation of siRNA-chitosan nanoparticles*

Formulations with different amino/phosphate (N/P) ratios were prepared by a self-assembly method while keeping the amount of siRNA constant (1.2 μg/mL). A solution of siRNA (5 μM, 6.65 μg/mL) was diluted with the necessary amount of sterile nuclease free water (5 Prime). Subsequently, the required amount of chitosan was added from a sterile filtered solution (0.1 mg/mL) during vortex mixing (1,200 rpm). The assembled nanoparticles were incubated for 30 min at room temperature before transfection. When assembling the nanoparticles for transfection at different doses, the particles were first made at the highest concentration and incubated for 30 min before serial dilution.

*Nanoparticle tracking analysis*

The nanoparticle concentrations were determined using nanoparticle tracking analysis (NTA) on a NanoSight LM10 (NanoSight) at a siRNA concentration of 500 nM. Measurements were performed in MQ water at room temperature using the viscosity of water in the calculations. The CCD camera was operated and video was captured with the software NTA 2.0. Video capture parameters such as the shutter value and recording gain and the analysis parameter detection threshold were set manually. The capture duration was set to 60 s and the temperature was recorded with a digital thermometer.

*Cell culture*

The immortalized rat endothelial cell line RBE4 [2] was kindly provided by Prof. Tore Syversen (Dept. of Neuroscience, NTNU). The cells were grown in alpha MEM (aMEM, Gibco, Invitrogen) supplemented with 10% FBS, 300 μg/mL G418 selection antibiotic (Sigma) and 1 ng/mL basic fibroblast growth factor (Invitrogen). When seeding cells for experiments, growth media supplemented with 100 U/mL of penicillin and streptomycin (PEST, Sigma) was used. The cells were cultivated on surfaces coated with rat tail type I collagen (BD Biosciences) at 37ºC in a humidified atmosphere with 5% CO_2_. Possible mycoplasma contamination was routinely monitored using the MycoAlert detection kit (Lonza).

*Transfection*

Cells were seeded in tissue culture wells 24 h prior to experiments in densities with approximately 50-75% confluency on the day of transfection. E.g. 7,500 cells in 100 μL growth medium were seeded in type I collagen coated 96-well plates (Corning). The nanoparticles assembled in water were diluted with an equal volume of Opti-MEM (Gibco, Invitrogen), supplemented with 270 mM mannitol (Sigma) and 20 mM HEPES (Sigma) for adjustment of the osmolarity to 300 mOsm/kg and the pH to 7.2. The formulations were not supplemented with FBS or antibiotics. Prior to adding the nanoparticles, the cells were washed and briefly incubated with 100 μL/well of Hank’s balanced salt solution (HBSS, Gibco, Invitrogen) at 37ºC and 5% CO_2_. Next, the HBSS solution was removed and 50 µl aliquots of nanoparticle formulation containing typically 0.067 μg (100 nM) siRNA were added to each well in 96-well plates. The formulations were removed after 5 h of incubation and replaced by 200 µL of growth media supplemented with PEST.

*Rhodamine 123 efflux assay*

Two days after transfection with anti-P-gp siRNA, cells were incubated with the P-gp substrate rhodamine 123 (R123, Sigma). A volume of 100 μL of a 10 μM R123 solution diluted in Opti-MEM was added to the cells. After 45 min of incubation, R123 was removed and replaced with growth medium. Two hours after removing the R123, cells were prepared for analysis by flow cytometry or CLSM.

*Doxorubicin delivery and metabolic activity assay*

One day after transfection with anti-P-gp siRNA, the RBE4 cells were added growth medium with concentrations of the P-gp substrate doxorubicin (Pharmacia) ranging from 0 to 5 μM. The cells were incubated with doxorubicin for two days before the effect on metabolic activity was measured using an Alamar Blue assay (Invitrogen). A volume of 10 µl of the Alamar Blue assay reagent that was diluted in 100 µl of growth medium without phenol red was added to the cells and the sample absorbances were measured 4 h after adding the assay reagent using a spectrophotometer (Molecular Devices) at 570- and 600-nm. The metabolic activities of the cells were determined from the fraction of Alamar Blue reagent that was turned over during a 4 h incubation period.

The evaluation of intracellular doxorubicin delivery by flow cytometry and CLSM was performed by incubating the cells in growth medium with 50 µM doxorubicin for 3 h. Afterwards, the samples were prepared for analysis by flow cytometry or CLSM.

*Flow cytometry*

Cellular uptake of siRNA, the R123 efflux and the doxorubicin delivery were evaluated using a Gallios flow cytometer (Beckman Coulter). The obtained data was analyzed and visualized using the Kaluza software package (Kaluza Flow Cytometry Analysis v1.1, Beckman Coulter).

The cellular uptake of siRNA was determined by transfection with Alexa-647 conjugated siRNA. After incubating with nanoparticles for 4 h, the cells were washed with PBS and further incubated with aMEM for 30 min to allow internalization of possible surface bound nanoparticles. Afterwards, the cells were incubated with heparin supplemented aMEM (1 mg/mL, Sigma) for 30 min to dissociate siRNA from possible remaining surface bound nanoparticles. The cells were then washed in PBS (Gibco, Invitrogen), trypsinized, resuspended in ice-cold PBS supplemented with 5% FBS and kept on ice until the time of analysis.

Intracellular R123 and delivery of doxorubicin was measured 48 h after transfection with anti-P-gp siRNA as previously described.

For each sample, 10,000 gated events were counted and a dot plot of forward scatter versus side scatter established a collection gate for cells to exclude cellular debris, dead and aggregated cells. The R123 and doxorubicin or Alexa-647 treated cells were excited using a 488 nm or 633 nm laser line, respectively. Emitted light was collected at FL1 (R123), FL2 (doxorubicin) or FL6 (Alexa-647) using 525/40 nm, 575/40 nm or 660/20 nm band pass filter, respectively. Untreated cells were analyzed to determine the normal levels of median fluorecence intensity (FI) from autofluorescence (uptake experiment), intracellular R123 or doxorubicin. The relative amounts of intracellular Alexa-647, R123 or doxorubicin were estimated from the median FI of the analyzed cells.

*Real-time quantitative reverse transcriptase PCR*

Knockdown of the ubiquitously expressed endogenous gene GAPDH (Glyceraldehyde-3-phospate dehydrogenase) and P-gp was measured at mRNA level using the ABI 7500 real-time PCR system (Applied Biosystems). The mRNA was harvested, and cDNA was synthesized and amplified using the Cells-to-C_T_ kit (Applied Biosystems) as described in the manufacturer’s protocol. Reverse transcription was performed at 37ºC for 60 min. Real-Time quantitative reverse transcriptase PCR (qRT-PCR) was performed using the following cycle conditions: 95ºC for 10 min, 40 cycles at 95ºC for 15 s, and 60ºC for 1 min. The primers that were used are described in table 2 (manuscript).

The primer efficiencies were determined using standard curves. The percentage of mRNA expression relative to untreated cells was calculated using the comparative C_t_ method, where the target sample was normalized to endogenous β-actin.

*GAPDH protein activity assay*

The effect of transfection with anti-GAPDH siRNA on the GAPDH protein activity was measured using the commercial available KDalert GAPDH assay kit (Ambion) according to the manufacturer’s protocol. The amounts of lysate and assay reagents were halved, and measurements were performed in half-area 96-well plates (Corning) at 615 nm using a spectrophotometer.

*CLSM*

RBE4 cells were seeded onto type I collagen coated 8-chamber microscopic slides (Ibidi) and transfected with Alexa-647 labelled siRNA or anti-P-gp siRNA with subsequent addition of R123 or doxorubicin as described previously. At the time of analysis, the cells were added 5 µg/mL of CellMask plasma membrane stain (Invitrogen) diluted in aMEM, as described in the manufacturer’s protocol. Live cells were examined using a LSM 510 (Carl Zeiss) confocal laser scanning microscope (CLSM) equipped with a c-Apochromat 40x/1.2 NA W corr objective. R123 and doxorubicin were excited using 488 nm argon, CellMask Orange was excited using 543 nm HeNe and Alexa-647 and CellMask Deep Red were excited using a 633 nm HeNe laser line. The emitted light was collected using 525/25 nm band pass (R123), 590/25 band pass (CellMask Orange and doxorubicin) or 650 nm long pass (CellMask Deep Red) filters. The acquired images had resolutions of 512x512 pixels.

*Statistical analysis*

The measurements were collected and expressed as mean values ± standard deviation (s.d.). Statistical differences between raw data were investigated using the SigmaPlot 11.0 software package with one-way ANOVA, in conjunction with a multiple comparison test (Holm-Sidak).

**Reference**

[1] Tømmeraas K, Vårum KM, Christensen BE, Smidsrød O (2001) Preparation and characterisation of oligosaccharides produced by nitrous acid depolymerisation of chitosans, Carbohydr R**e**s 333: 137-144.

[2] Roux F, Durieu-Trautmann O, Chaverot N, Claire M, Mailly P, et al. (1994) Regulation of

gamma-glutamyl transpeptidase and alkaline phosphatase activities in immortalized rat brain

microvessel endothelial cells. J Cell Physiol 159: 101-113.
